# High load of multi-drug resistant nosocomial neonatal pathogens carried by cockroaches in a neonatal intensive care unit at Tikur Anbessa specialized hospital, Addis Ababa, Ethiopia

**DOI:** 10.1186/2047-2994-1-12

**Published:** 2012-03-16

**Authors:** Birkneh Tilahun, Bogale Worku, Erdaw Tachbele, Simegn Terefe, Helmut Kloos, Worku Legesse

**Affiliations:** 1 Department of Pediatrics and Child Health, Addis Ababa University, Addis Ababa, Ethiopia; 2 School of Nursing, Addis Ababa University, Addis Ababa, Ethiopia; 3 Department of Microbiology, Addis Ababa University, Addis Ababa, Ethiopia; 4 Department of Epidemiology and Biostatistics, University of California, California, USA; 5 Tree Foundation Research Associate, Sarasota, USA

**Keywords:** *Blatella germanica*, Multi-drug resistant pathogens, Neonatal intensive care unit, Ethiopia

## Abstract

**Background:**

Cockroaches have been described as potential vectors for various pathogens for decades; although studies from neonatal intensive care units are scarce. This study assessed the vector potential of cockroaches (identified as *Blatella germanica*) in a neonatal intensive care unit setup in Tikur Anbessa Hospital, Addis Ababa, Ethiopia.

**Methods:**

A total of 400 *Blatella germanica *roaches were aseptically collected for five consecutive months. Standard laboratory procedures were used to process the samples.

**Results:**

From the external and gut homogenates, *Klebsiella oxytoca, Klebsiella pneumoniae, Citrobacter spp. Enterobacter cloacae, Citrobacter diversus, Pseudomonas aeruginosa, Providencia rettgeri, Klebsiella ozaenae, Enterobacter aeruginosa, Salmonella C1, Non Group A streptococcus, Staphylococcus aureus, Escherichia coli, Acinetobacter spp. and Shigella flexneri *were isolated. Multi-drug resistance was seen in all organisms. Resistance to up to all the 12 antimicrobials tested was observed in different pathogens.

**Conclusion:**

Cockroaches could play a vector role for nosocomial infections in a neonatal intensive care unit and environmental control measures of these vectors is required to reduce the risk of infection. A high level of drug resistance pattern of the isolated pathogens was demonstrated.

## Introduction

Cockroaches are one of the common-place pests widely distributed in public places, including hospitals, food industries and kitchens [1,2]. They feed on animal products, meat and grease, starchy food, sweets and unprotected materials [3]. The most abundantly distributed cockroach species is *Blatella germanica and *this is also the case as shown in an Ethiopian study [4-6].

The fact that cockroaches carry pathogenic bacteria has been reported for many years. Longfellow in 1912 showed that cockroaches carried pathogens, including *Proteus vulgaris, Staphylococcus aureus, Citreus *and *Bacillus *of the subtilis type [7]. Fungi of medical importance such as *Candida*: *Rhizopus, Mucor, Alternaria *and *Aspergillus *spp. were isolated from cockroaches in hospital wards and residential areas [8]. A recent study (2010) in central Tehran, Iran reported that at least 25 different species of medically important bacteria, the most frequent being *Klebsiella *spp., were isolated from *Blatella germanica *and *Periplaneta americanaI *collected from public hospitals and a residential house. Cockroaches from hospitals were more likely to be contaminated with medically important bacteria [9]. A study by Cotton *et al. *from South Africa compared pathogenic strains from cockroaches and those which colonize infants and found no difference [10].

Tachbele *et al. *studied the microbiology of cockroaches from hospitals and restaurants in Ethiopia, Addis Ababa, isolating four sero-groups of *Salmonella, Shigella, E. coli 0157, S. aureus *and *Bacillus cereus*. Most of the isolates were found in hospital cockroaches both from gut and external surfaces. However, they didn't find statistically significant differences in the distribution of potential pathogens between source location and body parts. Drug sensitivity was also determined in that study and more resistant cases were seen in hospitals [6].

In a study by Hsiu-Hua Pai *et al.*, 23 bacterial and 12 fungal isolates were found from *Blatella. germanica*. Multi-drug resistance with resistance to Ampicillin (13.7%-100%), Chloramphenicol (14.3%-71.4%), Tetracycline (14.3%-73.3%), and Trimethoprim-Sulfamethoxazole (14.3%-57.1%) was found in two gram-positive and five gram-negative bacteria [11].

There are insufficient data on the potential health impact of cockroaches in neonatal intensive care units. Yes, large numbers of cockroaches are encountered on beds, heaters, oxygen tubes and incubators of neonates at Tikur Anbessa Hospital in Addis Ababa. Cockroaches were also reported from an adult intensive care unit (ICU), calling for action to control these potential disease vectors and the nuisance they create in hospital settings. This article reports on the microbiology of cockroaches in the neonatal intensive care unit of Tikur Anbessa Hospital.

## Materials and methods

### Sample processing

*Blattella germanica *(Dictyoptera: Blattellidae) were randomly collected twice per week for five months from neonatal intensive care unit of Tikur Anbessa Hospital (TAH-NICU). During the study period no other species of cockroaches were encountered. The cockroaches were collected with hands wearing sterile-gloves from beds of newborns, heaters, oxygen cylinders and incubators and placed in sterile screw capped 250 ml jar. During each sampling session, 10 cockroaches were trapped randomly and pooled as one sample. Cockroaches caught whole and alive were included in the study and those Cockroaches which were dead, or showing missing body parts or gross recent contamination were excluded from further sample processing. Identification was made following the method by Burgess *et al. *[12].

Live cockroach samples were immediately transported to laboratory and killed using chloroform soaked cotton [6]. To wash the external body surface, they were soaked in 5 ml sterile normal saline for 2 minutes and the wash was taken as an external body homogenate sample. Specimens were immersed in 90% ethanol for 5 minutes to decontaminate their external surface and were allowed to air dry. Then they were washed with sterile normal saline to remove remnant ethanol. Finally their viscera were dissected using auto-clave sterilized entomological needles under a dissecting microscope to locate gut homogenates. Instruments were sterilized after every dissection. Their gut was then kept in 5 ml sterile normal saline for 5 minutes to produce a homogenate sample. One ml of homogenate sample was taken and inoculated into 9 ml of buffered peptone water (BPW) (OXOID, Basingstoke, UK) and incubated at 37°C for 24 hours [6,13].

### Bacteriological analysis

After the external and gut homogenates were prepared and incubated 24 hours in buffered peptone water (BPW), each one of the growth from BPW was inoculated in seven primary media (Mac-Conkey, Sheep Blood Agar, Chocolate agar, SS agar, Desoxycholate Citrate Agar-DCA, Xylose Lysine Deoxycholate- XLD and Mannitol Salt Agar-MSA) to grow most gram positive and negative bacteria [6,14]. From the plates, typical colonies of each organism were then transported to Trypton water broth (OXOID, Basingstoke, UK) for secondary enrichment at 37-42°C for 24 hours [6]. Serial dilutions were done throughout the study period.

Subsequently, organisms were presumptively identified based on colony character and color. Further characterization with gram stain and biochemical tests was done for each isolate. For the genus *Enterobacteriaceae*, biochemical tests, including lactose fermentation and reaction to indole, urea, mannitol, Tryptone Sugar Iron-TSI (H_2_S), Gas/glucose, Citrate Lysine decarboxylase - (LDC), malonate, oxidase, catalase and Lysine Iron Agar - (LIA) were done to identify the organism. These biochemical tests were used according to standardized identification protocols and flow sheets [15-20].

The lactose fermenting *Enterobacteriaceae*, including *Citrobacter spp., Enterobacter spp., Klebsiella spp., and Enterococcus *were identified by their reactions to indole, urea, mannitol, H_2_S, gas/glucose, citrate, lysine - (LDC), malonate and their motility [15-20].

*Salmonella *and *Shigella *were identified by streaking the secondarily enriched specimen on Xylose Lysine Deoxycholate (XLD) agar after 18-24 incubations at 37°C. They were distinguished with their characteristics on XLD agar [21]. Standard biochemical methods and BBL anti sera for sero-grouping were also used [22].

*Pseudomonas aeruginosa *and *Acinetobacter spp*. were differentiated by catalase, oxidase and malonate tests after the colony character and other biochemical tests. *Pseudomonas aeruginosa *was identified by its catalase, oxidase and malonate positivity [15-20].

*Staphylococcus spp*. was isolated from Mannitol Salt Agar- MSA (OXOID, Basingstoke, UK) and *staphylococcus aureus *was confirmed by both coagulase and catalase positivity [6,15,16,19,20]. *Streptococcus spp*. was identified from sheep blood agar by its beta-hemolytic nature and catalase negativity. On further testing with Optichin and Bacitracin tests, the isolate was a Non- group A *streptococcus *[23]. *Providencia rettgeri *was identified by all the listed biochemical testing and PAD positivity [17,18].

### In-vitro drug susceptibility testing

Susceptibility testing was done on Muller-Hinton agar plates following the standardized disk diffusion technique [24] with Oxoid drug disks(12): Ampicillin(Amp), (10 μg); Sulfamethoxazole (CTX), (25 μg); Amoxicillin/Clavulanic Acid (Aug),(30 μg); Chloramphenicol(Caf), (30 μg); Gentamicin (Gen),(10 μg for Enterobacteriaceae, 120 μg for enterococci); Amoxicillin(Amx), (20 μg); Cefotaxime (Cefo); (30 μg); Ceftriaxone (Ceft),(30 μg); Tetracycline(TTC); (30 μg), Doxycycline(Dox),(30 μg); Norfloxacin(Nrf), (10 μg); and Ciprofloxacin (Cip) (5 μg). Reading was in three formats: Sensitive(S), Intermediate (I), and Resistant(R) [25,26]. Because the number of Intermediate (I) readings were few (12), they were included in the sensitive group for the purpose of assessing the data [6].

### Statistical analysis

A comparison of isolation rates and proportion of drug resistance between body parts was made using Fisher's Exact test and Student's *t*-test. Level of significance was set at *P *< 0.05.

## Results

A total of 400 cockroaches were collected from the neonatal intensive care unit of Tikur Anbessa Hospital from mid-February 2011 to mid-July 2011. All the cockroaches were found to be *Blatella germanica*. There were a total of 80 homogenate samples (batch of 10 cockroaches pulled as one); 40 were gut homogenates and the other 40 were external homogenates, the samples were collected sequentially; the first sample was collected in February and the last in July. There were a total of 231 isolates of 12 species of pathogenic bacteria from the gut and external homogenates of these cockroaches. Growth of pathogenic bacteria was found in each of the gut and external homogenates. Almost all had a poly-microbial (more than one pathogen) growth.

There was no statistically significant difference in the level of isolation of pathogens from internal and external homogenates (*P-value = 0.48*). There was also no statistically significant difference in the isolation of the two spp. of each of *Klebsiella *spp. and *Citrobacter *spp. from external or gut homogenates (*P-value = *0.87 and 0.76 respectively). *Klebsiella *species were the most numerous isolates which were consistently isolated across time (94); the other isolates included *Citrobacter *species (55), *Enterobacter *species (35), *Pseudomonas *species (19), *Providencia *species (10), *Salmonella *species (4), *Non Group A streptococcus *(4) *Staphylococcus aureus *(4)*, E. coli *(3), *Acinetobacter spp*. (3), and *Shigella flexneri *(1) (Table 1).

**Table 1:** Isolates from external and gut homogenates of cockroaches collected from neonatal ICU of Tikur Anbessa Hospital, Addis Ababa, Ethiopia, July 2011

Isolate	External (%)	Gut (%)	Both*(%)	Total	*P*-value
*Klebsiella oxytoca*	20(18.7)	24(19.4)	11(25.6)	44(19)	**0.87**
*Klebsiella pneumoniae*	16(15)	24(19.4)	9(20.9)	40(17.4)	
*Citrobacter spp*	14(13)	17(13.7)	5(11.7)	31(13.4)	**0.76**
*Citrobacter diversus*	11(10.2)	13(10.5))	2(4.6)	24(10.4)	
*Enterobacter cloacae*	15(14)	13(10.5)	5(11.7)	28(12.2)	
*Pseudomonas aeruginosa*	10(9.3)	9(7.3)	5(11.7)	19(8.3)	
*Providencia rettgeri*	2(1.9)	8(6.4)	-	10(4.3)	
*Klebsiella ozaenae*	4(3.8)	3(2.4)	-	7(3.0)	
*Enterobacter aeruginosa*	5(4.7)	2(1.6)	1(2.3)	7(3.0)	
*Salmonella spp*	2(1.9)	4(3.2)	1(2.3)	6(2.6)	
*Non Group A streptococcus*	3(2.9)	1(0.8)	2(4.6)	4(1.7)	
*Staphylococcus aureus*	1(0.9)	3(2.4)	1(2.3)	4(1.7)	
*Escherichia coli*	1(0.9)	2(1.6)	1(2.3)	3(1.3)	
*Acinetobacter spp*	2(1.9)	1(0.8)	-	3(1.3)	
*Shigella flexneri*	1(0.9)	-	-	1(0.4)	

**Total**	**107(100)**	**124(100)**	**43(100)**	**231(100)**	**0.480**

* The number of similar isolates from each one of external and gut homogenates of individual batch of cockroaches

Drug sensitivity was done for all the isolates against 12 antibiotics (Table 2). Multidrug resistant pattern was seen in nearly all isolates. Resistance to penicillin (Ampicillin, Augmentin and Amoxacillin) was found to be high for all pathogens.

**Table 2:** In-vitro sensitivity testing of the isolates from neonatal ICU of Tikur Anbessa Hospital: Percentage of resistance of each pathogen to various antibiotics, July 2011

Isolate	**Num**. (N)	Amp (%)	Aug (%)	Amx (%)	Gen (%)	Ceft (%)	Cefo (%)	TTC (%)	Dox (%)	Caf (%)	CTX (%)	Nrf (%)	Cip (%)
*Klebsiella oxytoca*	**44**	100	98	100	98	91	86.4	72.7	63.6	91	98	41	52.3
*Klebsiella pneumonia*	**40**	100	100	100	97.5	100	100	95	95	62.5	97.5	52.5	67.5
*Citrobacter spp*	**31**	100	100	100	93.5	96.8	93.5	90.3	77.4	100	87.1	35.5	67.7
*Enterobacter cloacae*	**28**	100	100	100	93	93	93	71	82	89.3	93	43	68
*Citrobacter divursus*	**24**	95.8	87.5	91.7	91.7	83.3	79.2	75	70.8	83.3	79.2	29.2	37.5
*Pseudomonas aeruginosa*	**19**	100	100	100	79	89.5	89.5	89.5	89.5	89.5	84.2	5.2	5.2
*Providencia rettgeri*	**10**	100	100	100	100	100	100	100	90	90	100	10.0	60
*Klebsiella ozaenae*	**7**	100	100	100	85.7	100	100	85.7	85.7	83.3	100	43.0	57
*Enterobacter aeruginosa*	**7**	100	100	100	85.7	71.4	71.4	71.4	85.7	100	85.7	57	57
*Salmonella C*	**6**	100	100	100	100	100	100	83.3	83.3	100	66.7	0	0
*Non Group A streptococcus*	**4**	0	25	0	100	50	0	100	100	75	100	0	0
*Staphylococcus aureus*	**4**	100	75	75	50	50	50	25	25	0	25	50	50
*Escherichia coli*	**3**	100	100	100	100	100	100	100	100	33.3	100	66.7	66.7
*Acinetobacter spp*	**2**	50	100	50	50	50	50	100	0	50	50	50	50
*Shigella flexneri*	**1**	100	100	-	0	-	-	-	-	100	-	0	0

Amp = Ampicillin, Aug = Amoxicillin-clavulanate potassium, Amx = Amoxicillin Caf = Chloramphenicol, Gen = Gentamicin, Cefo = Cefotaxime, TTC = Tetracycline, CTX = Trimethoprim-sulfamethoxazole, Ceft = Ceftriaxone, Dox = Doxycycline, Nrf = Norfloxacin, Cip = Ciprofloxacin

More resistance to Gentamicin and Trimethoprim-sulfamethoxazole was found among gut isolates as compared to external isolates (96%vs 87% and 94%vs 83% respectively; *P*-value = 0.018 and 0.021 respectively) when compared with Fisher's Exact test for proportion resistant between body parts. There was no statistically significant difference in proportion of resistance between body parts for the other antimicrobials.

Over all, resistance to the fluoroquinolones (Norfloxacin and Ciprofloxacin) was found to be much lower. It was even lower for Norfloxacin than Ciprofloxacin. Cephalosporin (Cefotaxime and Ceftriaxone) resistance was found to be high for all the isolates but lower than for penicillin. The most frequent pattern of resistance was to all the 12 tested antimicrobials (Table 3).

**Table 3:** Pattern of resistance of the most common isolates from neonatal ICU of Tikur Anbessa Hospital, July 2011

Organism	No	Pattern of Resistance to Antimicrobials
*Klebsiella pneumoniae*	12	Amp, Aug, Caf, Gen, Amx, Cefo, TTC, CTX, Ceft, Dox, Nrf, Cip
	11	Amp, Aug, Caf, Gen, Amx, Cefo, TTC, CTX, Ceft, Dox
	9	Amp, Aug, Gen, Amx, Cefo, TTC, CTX, Ceft, Dox, Nrf, Cip
	4	Amp, Aug, Gen, Amx, Cefo, TTC, CTX, Ceft, Dox, Cip
	1	Amp, Aug, Caf, Gen, Amx, Cefo, TTC, CTX, Ceft, Dox, Cip
	1	Amp, Aug, Amx, Cefo, TTC, CTX, Ceft, Nrf, Cip
	1	Amp, Aug, Caf, Gen, Amx, Cefo, TTC, CTX, Ceft, Nrf
	1	Amp, Aug, caf, Gen, Amx, Cefo, TTC, Ceft, Dox
*Klebsiella oxytoca*	14	Amp, Aug, Caf, Gen, Amx, Cefo, TTC, CTX, Ceft, Dox, Nrf, Cip
	10	Amp, Aug, Caf, Gen, Amx, Cefo, TTC, CTX, Ceft, Dox
	5	Amp, Aug, Caf, Gen, Amx, Cefo, TTC, CTX, Ceft
	4	Amp, Aug, Caf, Gen, Amx, Cefo, CTX, Ceft
	3	Amp, Aug, Caf, Gen, Amx, Cefo, TTC, CTX, Ceft, Dox, Cip
	2	Amp, Aug, Gen, Amx, Cefo, TTC, CTX, Ceft, Dox, Cip
	2	Amp, Aug, Caf, Gen, Amx, CTX
	2	Amp, Aug, Caf, Gen, Amx, CTX, Ceft
	1	Amp, Aug, Caf, Gen, Amx, TTC, CTX, Dox, Nrf
*Enterobacter cloacae*	10	Amp, Aug, Caf, Gen, Amx, Cefo, TTC, CTX, Ceft, Dox, Nrf, Cip
	8	Amp, Aug, Caf, Gen, Amx, Cefo, TTC, CTX, Ceft, Dox, Cip
	3	Amp, Aug, Caf, Gen, Amx, Cefo, CTX, Ceft, Dox
	2	Amp, Aug, Caf, Gen, Amx, Cefo, TTC, CTX, Ceft
	1	Amp, Aug, Gen, Amx, Cefo, TTC, CTX, Ceft, Dox, Nrf, Cip
	1	Amp, Aug, Amx
	1	Amp, Aug, Caf, Gen, Amx, Cefo, TTC, CTX, Ceft, Dox
	1	Amp, Aug, Caf, Gen, Amx, TTC, CTX, Ceft, Dox
	1	Amp, Aug, Amx, TTC
*Citrobacter spp*	11	Amp, Aug, Caf, Gen, Amx, Cefo, TTC, CTX, Ceft, Dox, Nrf, Cip
	10	Amp, Aug, Caf, Gen, Amx, Cefo, TTC, CTX, Ceft, Dox, Cip
	4	Amp, Aug, Caf, Gen, Amx, Cefo, TTC, CTX, Ceft, Dox
	1	Amp, Aug, Caf, Gen, Amx, Cefo, Ceft,
	1	Amp, Aug, Caf, Amx,
	1	Amp, Aug, Caf, Gen, Amx, Cefo, TTC, CTX, Ceft, Dox, Cip
	1	Amp, Aug, Caf, Gen, Amx, Cefo, Ceft
	1	Amp, Aug, Caf, Amx, Ceft
	1	Amp, Aug, Caf, Gen, Amx, Cefo, CTX, Ceft
*Pseudomonas aeruginosa*	14	Amp, Aug, Caf, Gen, Amx, Cefo, TTC, CTX, Ceft, Dox
	3	Amp, Aug, Caf, Amx, Cefo, TTC, CTX, Ceft, Dox
	1	Amp, Aug, Caf, Gen, Amx, Cefo, TTC, Ceft, Dox,
	1	Amp, Aug, Caf, Gen, Amx, Cefo, TTC, CTX, Ceft, Dox, Nrf, Cip

## Discussion

Our findings show that there are many bacterial pathogens carried and harbored by cockroaches residing in the intensive neonatal care unit of Tikur Anbessa Hospital. These pathogens are common causes of neonatal nosocomial sepsis. Most isolated strains were multi-drug resistant. These findings indicate that cockroaches might be potential vectors for multidrug resistant bacteria pathogens in a neonatal intensive care unit set up and that their effective control and extermination is an important addition to infection prevention.

The "batching" of cockroaches used in this study might have hampered the identification of bacterial load of each cockroach in the batch. However, since the main purpose of the study was to identify the possible effect of cockroaches in NICU microbiology, the findings are important indicators of the need for urgent control of these potential vectors.

All cockroaches in this study were identified as *Blatella germanica*, a species widely associated with humans. This was also demonstrated by another study in Addis Ababa [6]. *Two Klebsiella *species were found to be the most frequent isolates in the current study. *Klebsiella *was also isolated from cockroaches by other researchers elsewhere in ICU and non-ICU settings [11,27,28]. This pathogen is the most common cause of neonatal sepsis in the same neonatal intensive care unit where we conducted our study [29]. The *Klebsiella *species isolated in our study were multidrug resistant; this was similarly documented by a study in TAH-NICU which demonstrated isolation of multidrug resistant *Klebsiella *from blood cultures of sick newborns^.^[26] However, the level of resistance of this pathogen for most antimicrobials was higher than most control studies [11,26,30,31].

*Citrobacter spp *was reported as important cause of neonatal nosocomial infections accounting for more than a third of all the identified infections in a study in Nepal [32]. The fact that cockroaches carry *Citrobacter *spp. has been shown by researchers elsewhere, though isolation was reported to be from house roaches [11,30]. In another study, the sources of nosocomial *Citrobacter *infection were indicated to be the hands of medical staff, water for injection, face masks, nasal prongs, stethoscopes, etc. [32]. However, the current study showed that *Citrobacter *species was the second most common isolate from cockroaches residing in the NICU. This finding is important as it points to one of the significant vectors for transmission of this emerging neonatal pathogen. The fact that the isolates showed a multi-drug resistant pattern is in agreement with a study by Khadka *et al. *[32]. In that study, the sensitivity of *Citrobacter *species to floroquinolones was shown to be higher, which is in agreement with our findings of good sensitivity for Norfloxacin and Ciprofloxacin.

*Pseudomonas aeruginosa *nosocomial infection is one of the devastating gram-negative neonatal infections. There are numerous studies which isolated *Pseudomonas *spp. from cockroaches in hospital settings [11,27,28,30]. *Pseudomonas *spp. is one of the common blood culture isolates from sick newborns in the TAH-NICU [33]. Hence, our findings strengthen the evidence that cockroaches may play a vector role for transmission of organisms to newborns. There was a multi-drug resistance pattern among all the isolates of *Pseudomonas *spp. Sensitivity for Floroquinolones (Ciprofloxacin and Norfloxacin) was found to be 95% as opposed to a 0%and 21% sensitivity to penicillin and gentamicin respectively. Similarly, resistance was reported to be 71.4%, 57.1% and 14.3% for Ampicillin, Gentamicin and Ciprofloxacin, respectively, by Hsiu-Hua Pai *et al. *[11] couldn't find NICU isolates of *Pseudomonas *from cockroaches, preventing comparison with our findings.

*Enterobacter spp*. neonatal bacteremia was found to be among the commonly encountered causes of neonatal sepsis by various bacteriologic studies [33-36]. Cockroaches as carriers of *Enterobacter *spp. were demonstrated by several studies elsewhere [11,27,28,30]. We showed that the most common *Enterobacter *species was *Enterobacter cloacae*. Finnstrom et al. [37] and Hervas et al. [38] reported outbreaks of *Enterobacter cloacae *sepsis in neonatal ICUs. Hence, isolation of a high load of *Enterobacter cloacae *from cockroaches sharing a room with neonates in this study is an important finding indicating the possible sources of infection. The current study also demonstrated the multi-drug resistant pattern of *Enterobacter spp; *a better sensitivity to floroquinolones (Ciprofloxacin and Norfloxacin) was observed, a pattern in agreement with the findings by Roy *et al. *[35].

*Salmonella spp*. was isolated from several health facilities in Ethiopia infested by cockroaches, including restraunts and hospitals [6,39,40]. But it has not been reported from the NICU of Tikur Anbessa Hospital. There are a considerable number of studies on the distribution and drug susceptibility pattern of *Salmonella *spp. in Ethiopia but there is no consistent finding on the predominant serotype. *Salmonella *microbiologic studies by Mache [41] and Asrat [31] showed a predominance of *Salmonella *serotype B, whereas a similar study by Beyene [42] revealed a higher number of serotype C isolates. Tachbele *et al. *[6] reported the most commonly isolated serotype from cockroaches as serotype B and didn't demonstrate isolation of serotype C. However, all the salmonella isolates in the current study were serotype C1. To resolve this discrepancy, a larger scale study may be required. There was a 100% sensitivity and resistance to Floroquinolones and Gentamicin, respectively. Resistance was also 100% for Penicillin and Chloramphenicol. A similar pattern was reported by control studies [43-45].

The finding of more resistance to Gentamicin and Trimethoprim-sulfamethoxazole among gut isolates in the current study was not shown in other cockroach researches for comparison.

Other smaller isolates of pathogenic organisms which similarly grew in other studies [6,14,45,46] included *Providencia retegerri, Staphylococcus aureus, E. coli, Acinetobacter spp*. and *Shigella flexneri. Non Group A. streptococcus *was isolated from both external and internal samples. This isolate was not described in other cockroach microbiologic studies elsewhere.

Based on the current and previous findings, the link between cockroaches and nosocomial infection has been established warranting provision of an effective and integrated pest management system. Such effective system of environmental control of cockroaches has to be implemented because of their notoriously resilient character and ability to readily infest diverse habitats [47].

## Conclusion

Our study documented a relatively high bacterial load and multi-drug resistant pattern, especially for the commonly used penicillin and aminoglycosides, compared to most other cockroach bacteriological studies in NICU settings and can be a useful addition to the existing body of literature on neonatal nosocomial infection control. The organisms carried and harbored by cockroaches are similar to those causing nosocomial neonatal sepsis. Cockroaches appear to be an important vector for multi-drug resistant neonatal nosocomial infections and neonatal intensive care units, urgently demanding their control through effective and integrated pest control systems.

## Competing interests

The authors declare that they have no competing interests.

## Authors' contributions

BT - developed the proposal, facilitated data collection and processing, did the analysis and write up. BW- conceived the initial idea, edited the proposal, and final paper. ET- edited the proposal and final paper and assisted in processing of cockroaches. ST- did the microbiology procedures. HK and WM extensively edited the final paper. All the authors read and approved the final paper.
